# A xylose-stimulated xylanase–xylose binding protein chimera created by random nonhomologous recombination

**DOI:** 10.1186/s13068-016-0529-7

**Published:** 2016-06-06

**Authors:** Lucas Ferreira Ribeiro, Jennifer Tullman, Nathan Nicholes, Sérgio Ruschi Bergamachi Silva, Davi Serradella Vieira, Marc Ostermeier, Richard John Ward

**Affiliations:** Johns Hopkins University, Baltimore, MD USA; Institute for Bioscience and Biotechnology Research, Rockville, MD USA; Departamento de Bioquímica e Imunologia, FMRP-Universidade de São Paulo-USP, Ribeirão Preto, SP Brazil; Universidade Federal do Rio Grande do Norte, Natal, Brazil; Laboratório Nacional de Ciência e Tecnologia do Bioetanol-CTBE, Centro Nacional de Pesquisa em Energia e Materiais (CNPEM), Campinas, SP Brazil; Departamento de Química, Faculdade de Filosofia, Ciências e Letras de Ribeirão Preto, Universidade de São Paulo, Av. Bandeirantes, 3900, Ribeirão Preto, SP 14040-901 Brazil

**Keywords:** Enzyme engineering, Xylanase, Allosteric regulation, Nonhomologous recombination, Directed evolution, Molecular dynamics simulation

## Abstract

**Background:**

Saccharification of lignocellulosic material by xylanases and other glycoside hydrolases is generally conducted at high concentrations of the final reaction products, which frequently inhibit the enzymes used in the saccharification process. Using a random nonhomologous recombination strategy, we have fused the GH11 xylanase from *Bacillus subtilis* (XynA) with the xylose binding protein from *Escherichia coli* (XBP) to produce an enzyme that is allosterically stimulated by xylose.

**Results:**

The pT7T3GFP_XBP plasmid containing the XBP coding sequence was randomly linearized with DNase I, and ligated with the XynA coding sequence to create a random XynA–XBP insertion library, which was used to transform *E. coli* strain JW3538-1 lacking the XBP gene. Screening for active XBP was based on the expression of GFP from the pT7T3GFP_XBP plasmid under the control of a xylose inducible promoter. In the presence of xylose, cells harboring a functional XBP domain in the fusion protein (XBP+) showed increased GFP fluorescence and were selected using FACS. The XBP+ cells were further screened for xylanase activity by halo formation around xylanase producing colonies (XynA+) on LB-agar-xylan media after staining with Congo red. The xylanase activity ratio with xylose/without xylose in supernatants from the XBP+/XynA+ clones was measured against remazol brilliant blue xylan. A clone showing an activity ratio higher than 1.3 was selected where the XynA was inserted after the asparagine 271 in the XBP, and this chimera was denominated as XynA–XBP271. The XynA–XBP271 was more stable than XynA at 55 °C, and in the presence of xylose the catalytic efficiency was ~3-fold greater than the parental xylanase. Molecular dynamics simulations predicted the formation of an extended protein–protein interface with coupled movements between the XynA and XBP domains. In the XynA–XBP271 with xylose bound to the XBP domain, the mobility of a β-loop in the XynA domain results in an increased access to the active site, and may explain the observed allosteric activation.

**Conclusions:**

The approach presented here provides an important advance for the engineering enzymes that are stimulated by the final product.

**Electronic supplementary material:**

The online version of this article (doi:10.1186/s13068-016-0529-7) contains supplementary material, which is available to authorized users.

## Background

Xylanases (endo-1,4-xylanase, EC 3.2.1.8) are the key enzymes involved in hemicellulose degradation, hydrolyzing the β-1,4-glycosidic bonds between xylose residues in xylan. Based on the amino acid sequence similarity and 3D-structural homology, xylanases have been grouped in glycoside hydrolase (GH) families GH5, GH8, GH10, GH11, and GH43 (CAZy; http://www.cazy.org/) [1, 2]. More recently, xylanolytic activity in members of the GH30 [3] and GH44 [4] families has also been demonstrated. The GH11 xylanases have been extensively studied as model enzymes for understanding the molecular basis for glycosidase mechanisms in general and also have numerous commercial applications in the paper, food and biofuel industries [1].

The saccharification of lignocellulosic material by xylanases and other glycoside hydrolases is generally conducted at a high concentration of solids to minimize water use in industrial processes, and these conditions inevitably result in the accumulation of high concentrations of the final reaction products. These reaction products frequently inhibit the enzymes that are used in the saccharification process [5–7], and application of classical enzymology tools is not always sufficient to circumvent this problem. For example, in the case of overcoming the inhibition of enzymatic activity against an insoluble substrate by a soluble competitive inhibitor, the addition of more substrate may not result in the recuperation of the reaction velocity since a polymeric substrate with a slow rate of diffusion is not capable of effectively competing for the active site with smaller molecules having higher diffusion rates. Thus, overcoming product inhibition is a prerequisite for the practical use of enzymes in bioprocesses, and a promising approach in the development of biocatalysts with increased efficiency through positive modulation of their enzymatic activity.

Bioengineers have been able to create allosteric couplings between biologically unrelated protein domains using different approaches that are generally based on in vitro protein evolution strategies [8–12]. The majority of these studies have the objective of creating protein switches, where proteins show either active (“on”) or inactive (“off”) states in response to the binding of an effector molecule. However, in the context of biorefineries aimed at biomass saccharification, it is more important for the enzymes to be active throughout the entire process and switching behavior is focused on maintaining the enzyme in the active state. To improve biocatalysis for biomass conversion we have recently used a semi-rational protein design strategy to insert a xylanase GH11from *Bacillus subtilis* (XynA) into a xylose binding protein (XBP) to create chimeric enzymes showing allosteric stimulation of catalytic activity by xylose [13]. This demonstrates the possibility of engineering lignocellulolytic enzymes that are stimulated by a specific effector through the combination of a binding domain with a catalytic domain. We have expanded this study using random nonhomologous recombination, and here we present the creation of a chimeric enzyme between xylanase and XBP that presents improved catalytic efficiency in the presence of xylose.

## Results and discussion

### Random insertion library construction and screening for xylose stimulation

We have recently demonstrated that it is possible to use a protein engineering approach commonly applied in the construction of protein switches to create xylanases stimulated by xylose, thereby increasing the catalytic efficiency of these enzymes in conditions under which the enzymatic activity is usually inhibited, such as high concentrations of xylose [13]. In the previous study, the xylanase was inserted into XBP at previously defined structural positions using a semi-rational structure guided strategy. Here, with the objective of finding new configurations between these two domains that enable greater intra molecular communication, the xylanase was inserted in a random manner into the XBP.

**Fig. 1:**
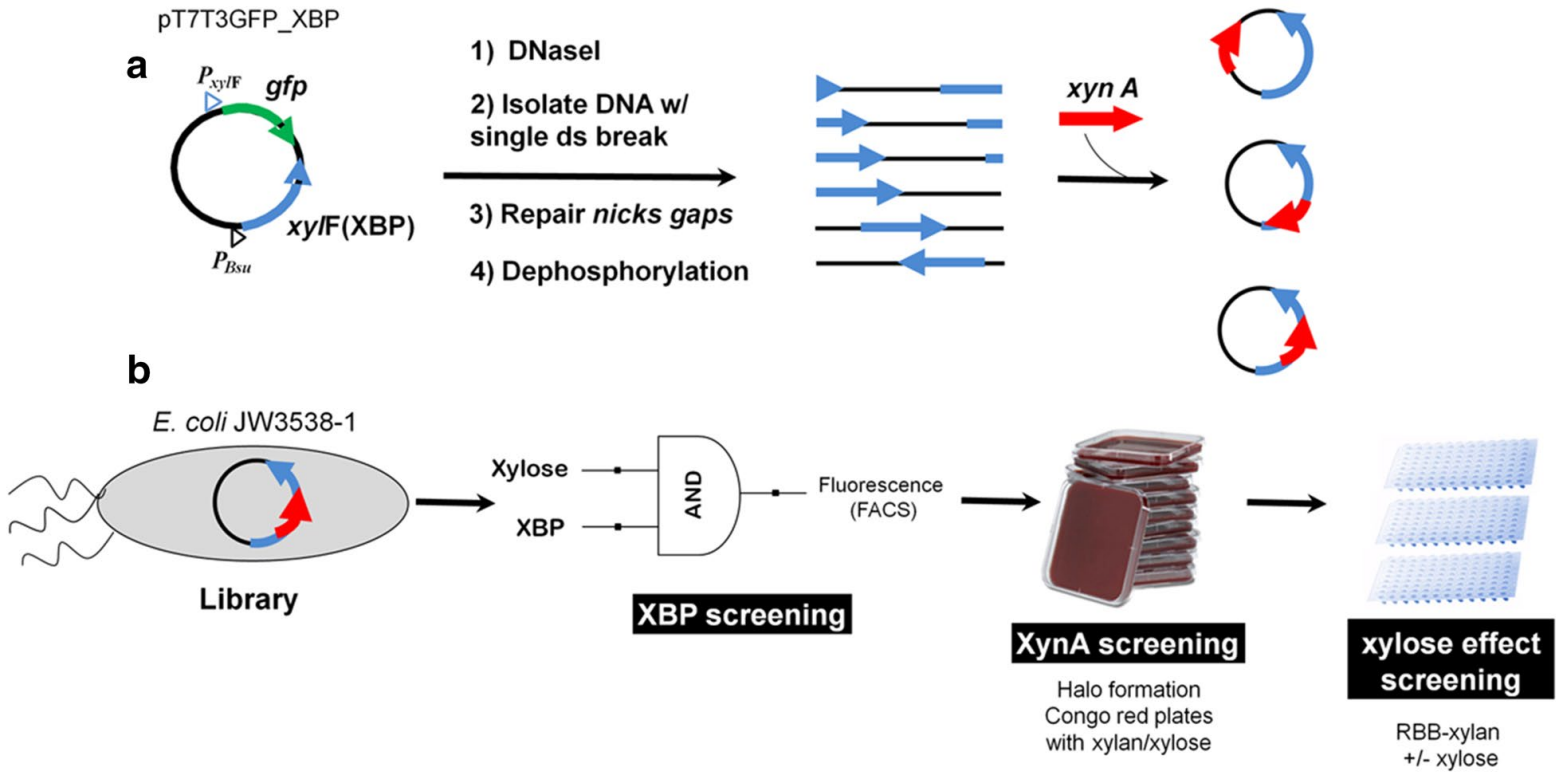
Creation and screening of a xylose-stimulated xylanase chimera. **a** The pT7T3GFP_XBP plasmid containing the xylose inducible GFP gene and XBP gene (xylF) under a constitutive promoter was randomly linearized and ligated to a xylanase gene (xynA from *B. subtilis*), resulting in a library of random insertions of xylanase into this plasmid. **b** After transformation of *Escherichia coli* with genomic XBP knocked out, the library was screened for functional XBP using a gene circuit for increased fluorescence in the presence of xylose. The XBP+ clones were separated by cell sorting and were plated on selective TB-agar media, supplemented with xylan in the presence of xylose, and the clones expressing xylanase activity (XynA+) were identified by the formation of halos on solid agar xylan plates after staining with *Congo red*. Xylose-stimulated catalytic activity in supernatants of XBP+/XynA+ clones were assayed for hydrolysis of RBB-xylan in the presence and absence of xylose

Random insertion libraries can be created using dilute concentrations of DNase I to generate a single random double-stranded break in the plasmid-borne acceptor gene [10, 14]. Thus, the *Escherichia coli* XBP was cloned into plasmid pT7T3GFP to create plasmid pT7T3GFP_XBP that was used as the target for insertion of the xylanase gene (Fig. 1a). The insertions were made at sites created using DNase I under controlled conditions such that each vector copy was linearized by random DNA cleavage and ligated to a XynA coding sequence, resulting in a library of random insertions of xylanase at all nucleotide positions of the plasmid. This “naïve” library was comprised of 2.8 × 10^5^ transformants, of which approximately 60 % contained the xylanase insert. Sequencing of the plasmid DNA from randomly selected colonies revealed that the insertions were distributed throughout the plasmid, and deletions varying from 50 to 543 bp were observed in all the sequenced clones (data not shown). Other studies using DNase I to create random insertion libraries and random circular permutation libraries also have found deletions distributed along the acceptor sequence, and contribute to the sequence variability of the library, potentially generating relevant diversity for the creation of new properties in the chimeric proteins [10, 14–16].

**Fig. 2:**
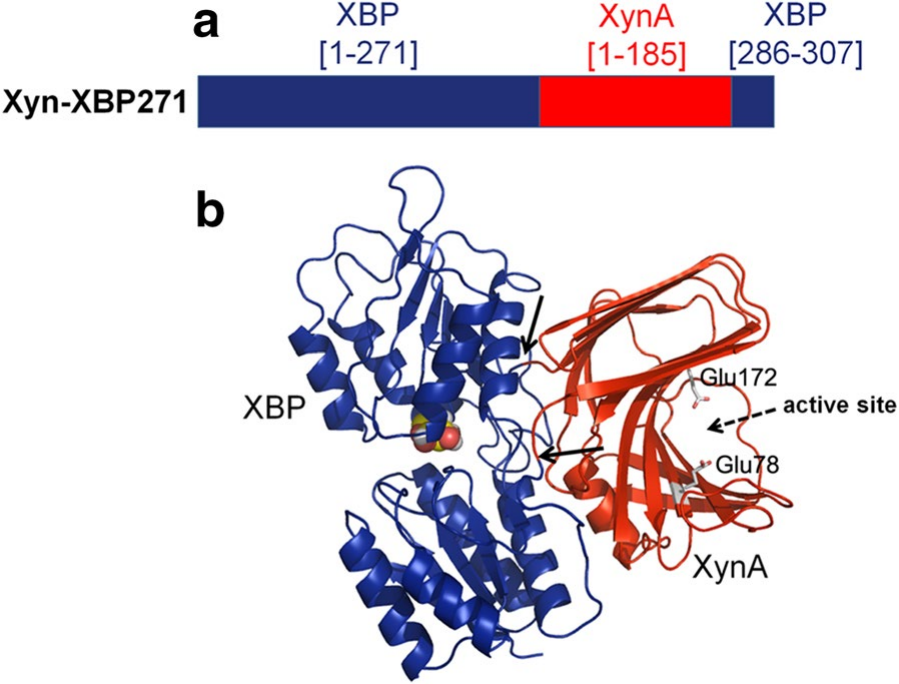
XynA–XBP chimera presenting the greatest positive modulation by xylose. **a** The sequence of the XynA–XBP271 chimera. Regions derived from XBP are shown in *blue* and those from XynA are shown in *red*. The number in *parentheses* indicates the amino acid number of the mature parental proteins. **b** Ribbon representation of the final model from molecular dynamics simulations of the XynA–XBP271 chimera. The structure of the xylose-bound XBP is shown in *blue*, and that from XynA is shown in *red*. The *arrows* indicate the xylanase insertion points in the XBP molecule. The active site and catalytic residues Glu78 and Glu172 in the xylanase domain are indicated

Random plasmid cleavage using DNase I generated insertion sites outside the XBP coding sequence, including frame-shifted and inverted insertions, generating a significant fraction of non-functional constructs, and therefore the library creation was combined with a powerful screening system (schematized in Fig. 1b). The initial selection for functional XBP domains by FACS was performed using a gene circuit in which the positive regulation of GFP by xylose was used to detect XBP activity. The promoter regulating the xylF gene encoding the XBP (*xylF*) is induced by xylose in a concentration-dependent response [17]. Since the main route for xylose uptake by *E. coli* is via XBP, *xylF* induction indicates that the cell harbors a functional XBP. The xylose-uptake deficient *E. coli* JW3538-1 (in which the genomic copy of *xylF* has been knocked out) was transformed with the random insertion library and screened for XBP activity using a gene circuit in which expression of the GFP in pT7T3GFP_XBP is under the control of the xylose inducible promotor of the *xylF* (PxylF). In the presence of xylose, increased GFP fluorescence was detected only in those cells harboring a functional XBP [17]. To define the upper and lower limits for detection of the XBP+ population, the vector without a copy of XBP (pT7T3GFP) was used as a negative control and the vector with a copy of the parental XBP (pT7T3GFP_XBP) was used as a positive control (see Additional file 1). A total of 202,151 events detected by FACS were analyzed and the sorted cells were plated on solid selective agar media, from which 2.09 × 10^5^ colonies were obtained. From this population, 2112 clones were analyzed for xylanase activity in the presence of d-xylose by formation of halos on solid agar plates containing xylan after staining with Congo red, (Fig. 1b; Additional file 1), Of these, ~15 % of the colonies formed halos and were classified as XBP+/XynA+ . These colonies were inoculated into deep well plates, and xylanase activity in the supernatant was measured both in the presence and absence of xylose (Fig. 1b). Of the 288 XBP+/XynA+ clones analyzed, 4 % (10 clones) showed a greater than 10 % increase in xylanolytic activity in the presence of xylose (see Additional file 1). The plasmid DNA isolated from the clone that showed the largest positive modulation (35 %) in the presence of xylose was sequenced. In this clone, the xylanase coding sequence was inserted after the N271 in the XBP. In addition, this chimeric construct also contained a deletion of 14 residues (272–285) in the XBP, corresponding to a β-strand in the C-terminal region of the XBP. This clone was denominated as XynA-XBP271 (Fig. 2).

### Biochemical and kinetic characterization

**Fig. 3:**
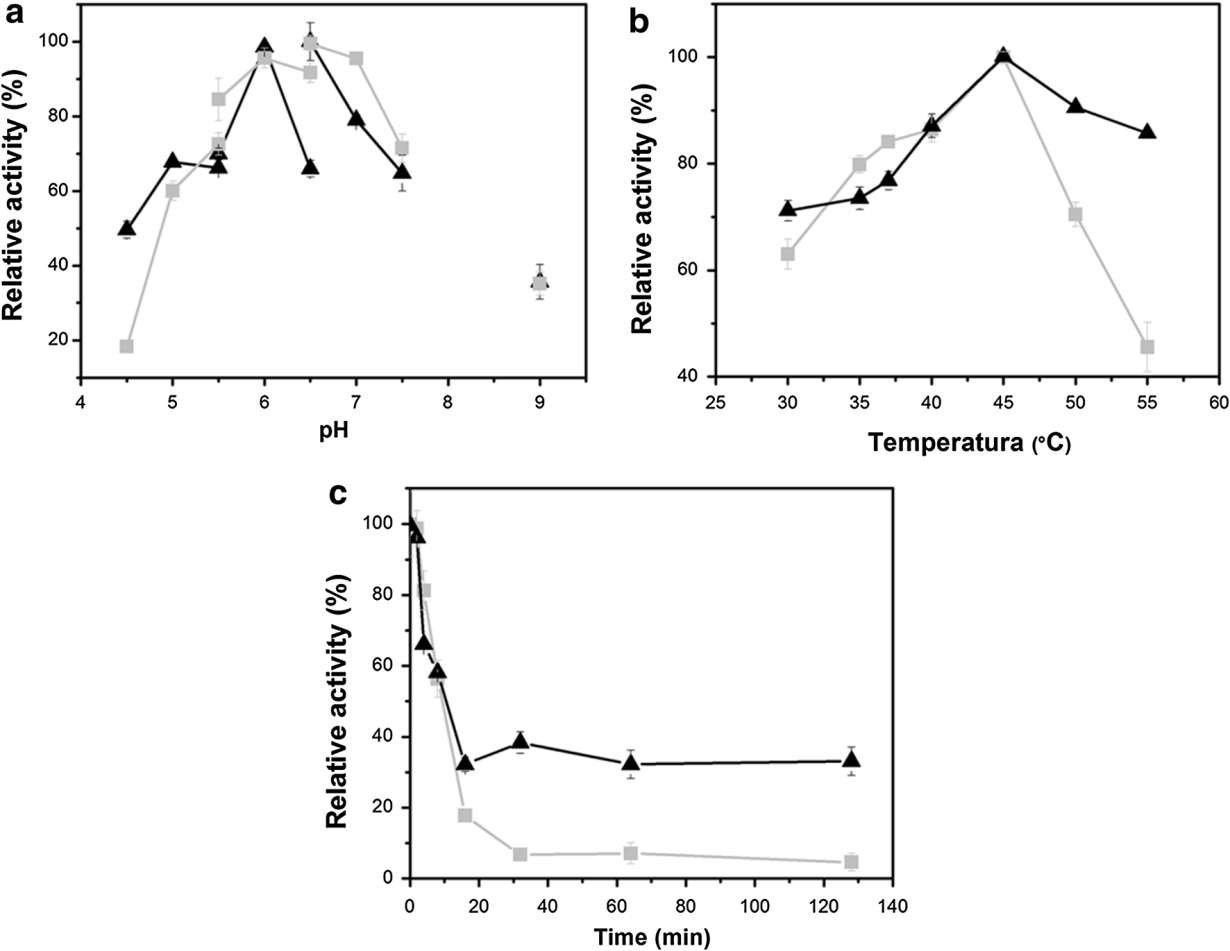
Biochemical characterization of the xylanase activity of the XynA and the XynA-XBP271 chimera. **a** The effect of pH. The interconnected points represent the following buffers at a final concentration of 50 mM: acetate (pH 4.5–5.5); phosphate (5.5–6.5), MOPS (pH 6.5–7.5) and arginine-NaOH (pH 9.0). **b** The effect of temperature, and **c** thermal inactivation at 55 °C. The *symbols* in all graphs are as follows: *filled square* parental XynA; and *filled triangle* XynA–XBP271chimera. *Error bars* show the mean ± sd

The parental proteins XynA and XBP together with the Xyn–XBP271 chimera were expressed, purified (Additional file 2), and submitted to biochemical and kinetic characterization. As shown in Fig. 3a, the optimal activity for the hydrolysis of RBB-xylan in MOPS buffer was observed at pH 6.5 both for the parental XynA and for the chimera. Phosphate buffer resulted in a decrease of ~40 % in the activity of the chimeric enzyme at this pH. In pH 4.5 acetate buffer the chimeric enzyme showed greater activity relative to the parental xylanase (XynA = 18 ± 2 %; Xyn–XBP271 = 49 ± 3 %). The chimeric enzyme showed a 48 % increase in activity over the pH range 4.5–7.5 as compared to the parental XynA.

Both the chimeric enzyme and the parental xylanase showed maximum activity at 45 °C (Fig. 3b). However, the chimeric enzyme maintained around 85 % activity at 55 °C, while the parental enzyme showed an abrupt decrease (greater than 50 %) in activity at this temperature. The thermostability of XynA and Xyn–XBP271 was measured at 55 °C (Fig. 3c). Under these conditions, activity of the parental XynA was reduced by 93 % after 32 minutes. In contrast, the chimeric enzyme retained 61 % activity after 32 minutes and 33 % activity after 128 minutes. The Xyn–XBP271 was shown to be stable with low aggregation of poorly folded proteins observed under the expression conditions used. Previous studies have shown that, in common with other periplasmic binding proteins, XBP has a broad optimal pH range, from 5.0 to 8.5, and does not suffer alterations in xylose binding capacity in this range [18]. Additionally, XBP has significant thermostability, with a half-life (t_1/2_) at 80 °C of 5 minutes [18], which is greater than the t_1/2_ of 2.2 minutes estimated for XynA at 70 °C [19]. These characteristics show the pH and temperature compatibility of XBP with the xylanase domain, and in addition suggest a possible thermal stabilization of the catalytic domain by the fusion with XBP. Recent studies show that the insertion of a less stable domain in a more thermostable domain may be an important strategy to increase the thermostability of the less stable protein [19–22]. Furthermore, it is noteworthy that the fusion with XBP involves both the N- and C-terminal regions of the xylanase, and alterations in both these regions are known to influence thermostability of the XynA [23, 24].

The kinetic properties of the purified parental and chimerical enzymes were compared, and Table 1 presents the values for the apparent dissociation constant (*K*_RBB-Xylan_), catalytic efficiency (*k*_cat_/*K*_RBB-Xylan_) and *n*_H_ (Hill coefficient). The value of the *K*_RBB-Xylan_ of the parental xylanase was similar to that of previously reported [13]. An approximate fivefold decrease was observed in the *K*_RBB-Xylan_ value for the XynA–XBP271 in comparison with the parental XynA, although no changes were observed in this parameter after the addition of xylose. The values for the catalytic efficiency (*k*_cat_/*K*_RBB-Xylan_) of XynA in the presence of xylose showed a ~15 % decrease (Table 1), as previously reported for this enzyme [13]. However, the observed catalytic efficiency values for the XynA activity in this study were lower than the previously reported values [13], due to differences in the methodology for determination of the enzymatic activity between the two studies. In the absence of xylose, the catalytic efficiency of the chimeric enzyme was ~2× greater than for the parental XynA. Furthermore, in the presence of xylose, this increase was even more pronounced, reaching ~3×. The catalytic efficiency of the XynA–XBP271 in the presence of xylose was 50 % greater than that observed in the absence of xylose. The chimeric enzyme also presented positive substrate cooperativity with a Hill coefficient (*n*_H_) > 2.0. Furthermore, comparison of the changes observed in the kinetic properties resulting from domain insertion for two XynA–XBP chimeras derived from a previous semi-rational fusion study (denoted as XynA-XBP2091A and XynA-2621B) [13] reveals an altered *K*_RBB-Xylan_value for XynA–XBP271 whereas for the XynA–2621B the main property affected was the *k*_cat_ (twofold higher than XynA parental). Moreover, both chimeras showed a similar modulation level in response to xylose (50 % increase). In the presence of xylose, both the chimeric proteins presented a higher catalytic efficiency in comparison with parental xylanase (threefold increase for XynA–XBP271 and 2.5-fold increase for XynA–2621B). Therefore, these results suggest that both substrate affinity and turnover number can be changed according to the configuration between XynA and XBP domains after fusion.

**Table 1:** Kinetic parameters of the chimeric enzyme compared with the parental enzyme

	Parental XynA		Chimera	
	−xylose	+xylose	−xylose	+xylose
*K*_RBB-Xylan_^a^	1.7 ± 0.2	1.4 ± 0.1	0.33 ± 0.2	0.33 ± 0.1
*k*_cat_/*K*_RBB-Xylan_^b^	0.47 ± 0.03	0.39 ± 0.02	0.86 ± 0.08	1.29 ± 0.03
*n*_H_	1.0	1.3	2.4	2.2

Data represent the mean ± SD

^a^mg mL^−1^

^b^mL mg^−1^ s^−1^

### Enzyme activity against sorghum stover

**Fig. 4:**
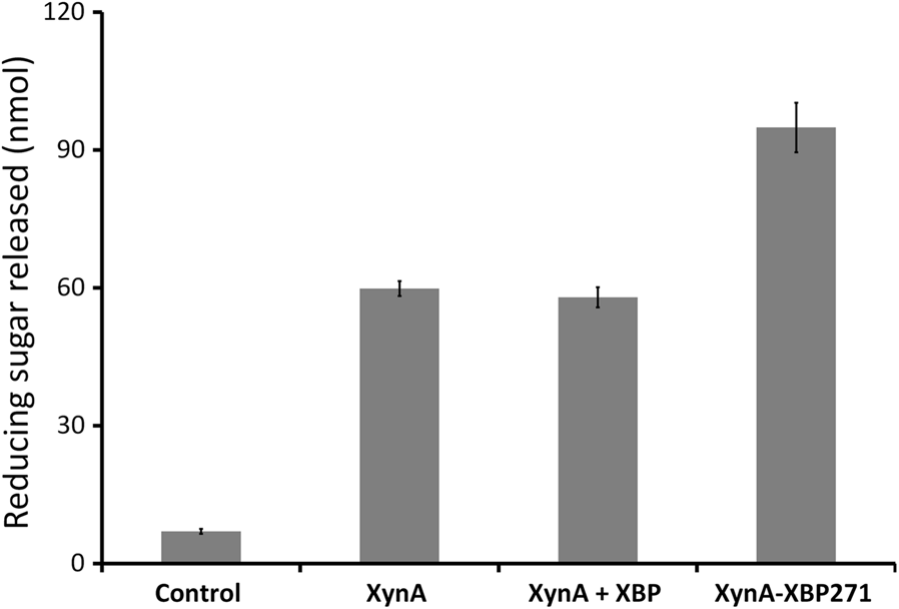
Reducing sugar release from ground sorghum stover by the XynA and the XynA–XBP271 chimera. The natural lignocellulose substrate was treated with the parental xylanase (XynA), an equimolar mixture of XynA and XBP (XynA + XBP), or the XynA–XBP271chimera. The control was treated under the same conditions but without enzyme. The result shows total reducing sugar released in nanomoles. Triplicate assays were performed in 100 mM MOPS buffer (pH 6.5) at 40 °C for 15 h. *Error bars* show the mean ± sd

The activity of the chimeric enzyme against a natural lignocellulose substrate was evaluated by measuring the total reducing sugar released after treatment of ground sorghum stover with the parental enzyme (XynA), with an equimolar mixture of the XynA + XBP, and with the XynA–XBP271 chimera (Fig. 4). As expected, since XBP has no catalytic activity, the effect of XynA alone was essentially the same as a mixture of the XynA + XBP. In contrast, the amount of reducing sugar released from sorghum stover by the chimeric enzyme was 37 % greater than the parental XynA enzyme, confirming the increased efficiency of the chimeric enzyme against natural biomass. Although the difference in enzymatic activity between the engineered and parental enzymes was lower with the sorghum stover than with RBB-xylan, this may be due to the complexity and/or architecture of the natural lignocellulosic substrate, which contains 29.1–31.3 % cellulose, 23.9–28.2 % hemicellulose and 7–7.3 % lignin [25], forming a complex and recalcitrant matrix with an effective pore size that is likely to hinder enzyme access to the xylan substrate. Furthermore, since the XynA releases mainly xylobiose, xylotriose and only a small amount of xylose [26, 27], it is possible that in the assay conditions used XynA–XBP271 was not in a fully stimulated state. Thus, to increase xylose release from a natural substrate it is necessary to combine xylanase GH11 with a β-xylosidase activity [28].

### Molecular dynamics simulations

**Fig. 5:**
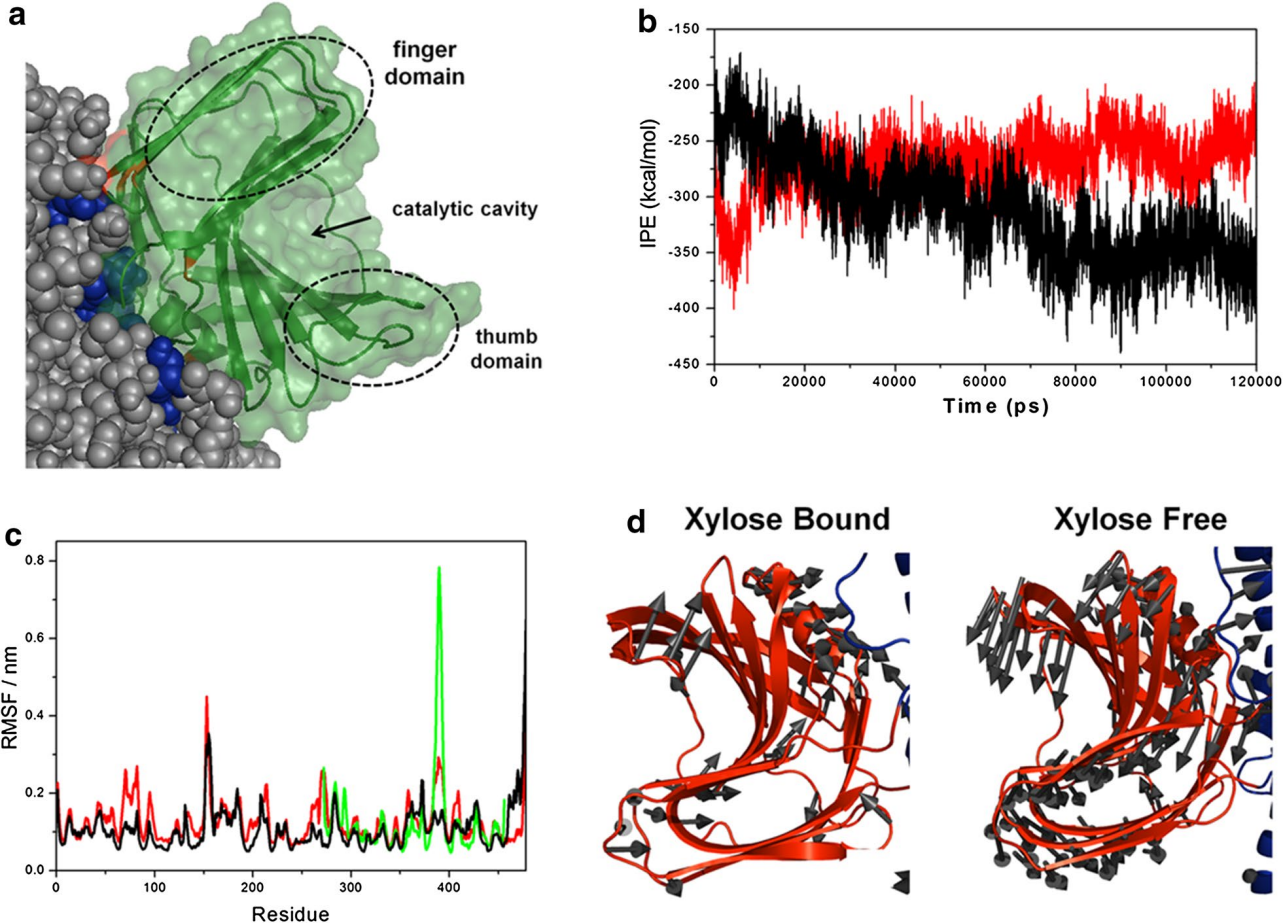
MD simulations of the XynA–XBP271 chimera. **a** Representation of the final xylose-bound XynA–XBP271 structure after molecular dynamics simulations. Details of the inter-domain interface are illustrated, where the XynA domain is shown as a cartoon and transparent surface and XBP is shown in a space-filling representation. Hot-spot residues at the protein–protein interface between XBP and XynA are shown in *blue* (XBP residues) and *red* (XynA residues). The catalytic site of the XynA is located between the palm and fingers domains, and access to the active site cleft is determined by the orientation of the thumb domain. **b** Comparison of the inter-domain interaction potential energy (IPE) as a function of simulation time in the presence (*black line*) and absence (*red line*) of xylose in the chimera enzyme. **c** Local fluctuations (RMSF) of parental XynA (*green line*) and the XynA–XBP271 chimera in the presence (*black line*) and absence (*red line*) of xylose. **d** Essential dynamics of the chimeras represented by *arrows* on an equilibrated representative 3D structure from MDS. The *arrows* indicate the direction of the local movements of the polypeptide chain in the XynA domain in chimera in the presence and absence of xylose

Molecular dynamics simulations (MDS) suggested the formation and stabilization of a protein–protein interface between the two domains of the XynA-XBP271 chimeric enzyme, both in the presence and absence of xylose (Fig. 5a, b). Similar interdomain protein–protein interfaces were also detected in MDS of the two XynA–XBP chimeras recently described by Ribeiro et al. [13]. The interaction potential energies (IPE) of the interdomain protein–protein interfaces in the XynA–XBP271 in the presence and absence of xylose were calculated and the results are presented in Fig. 5b. Fluctuation of IPE values overtime is a reliable parameter for protein–protein interface analysis [29, 30], and the average IPE computed for xylose-bound and free chimeras are −311.78 ± 43.74 and −270,74 ± 24.79 kcal/mol, respectively. Comparison of the interaction energies of the interfaces in the XynA–XBP271 chimera with those for the 2091A and 2621B chimeras [13], revealed that the interface in the xylose bound XynA–XBP271 chimera is approximately 120 kcal/mol more stable than the similar interface that is present in the xylose bound XynA-2621B chimera (the most stable interface reported previously [13]). Therefore, the optimization of the interdomain interface as a consequence of binding xylose is strongly dependent on the position in which the xylanase is fused to the XBP. This protein interface in the XynA–XBP271 chimera was investigated in detail using computational alanine scanning to identify the residues that contribute to the stabilization of the interdomain interface. An increase in $\Delta \Delta G_{\text{binding}}$ indicates a destabilization of the interface after introduction of the alanine mutant at the given position and when the $\Delta \Delta G_{\text{binding}}$ values after mutation exceed 1 kcal/mol, the position is considered to be a *hot spot* for stabilization of the protein–protein interface. For the chimera in the absence of xylose, 35 residues were identified with 8 hot spots, with $\Delta \Delta G_{\text{binding}}$ values varying from 1.1 to 2.9 kcal/mol (see Additional file 3). For the chimera in the presence of xylose, 52 residues were identified with 17 hotspots having $\Delta \Delta G_{\text{binding}}$ values varying from 1.2 to 4.4 kcal/mol. The residues E111 (in the XBP domain), S293 and F319 (in the XynA domain) make significant contribution to the stability of the interface in the xylose bound chimera, with values for ΔΔG_binding_ > 3.5 kcal/mol. These residues may be considered the principal hot spots at the interdomain interface, and are of potential importance for the protein–protein interface stabilization.

Polypeptide flexibility was quantitatively analyzed using the root mean square of the fluctuation (RMSF) per residue, using the alpha carbon atoms of each amino acid for the calculations. The calculated RMSF (Fig. 5c) reveals differences in flexibility in the alpha carbon positions when comparing the parental xylanase with the xylanase domain in the XynA–XBP271 chimera in the presence and absence of xylose. Significant fluctuations are observed in the thumb region of the xylanase (between residues 380 and 395) in all structures, and were highest in the parental XynA, less in the XynA–XBP271 without xylose, and lowest in the XynA–XBP271 in the presence of xylose. These results indicate that the fusion of XBP to the xylanase significantly reduces the flexibility of the thumb region, and that thumb flexibility is further reduced by the binding of xylose to the XBP.

Analysis of the essential movements of the XynA–XBP271 chimera can be derived from MDS results, and can provide deeper insights as to the structural and functional differences observed in the presence or absence of xylose. The essential movements can be visualized using a representative structure from MDS in which arrows indicate the direction of atomic movements to highlight large-scale protein motions over the time scale of the simulation (Fig. 5d). Different movements in the xylanase domains were observed in the parental enzyme and the chimera. Previous simulation studies have shown a transition between “open” and “closed” conformations regulated by opposed movements of the thumb and finger domains in the XynA [31, 32]. However, this unique transition was not present in xylanase moiety in the chimeric enzyme, where a twisting movement of the thumb domain relative to the beta-structures in the finger domain was observed. Well-defined thumb movements were observed both in the presence and absence of xylose, however, in the absence of xylose the catalytic cleft in the XynA became more exposed to solvent.

The volume of the active site of the xylanase was calculated using the EPOSPB tool (http://gepard.bioinformatik.uni-saarland.de/software/epos-bp) and the average result obtained for the XynA–XBP271 chimera was 1150 Å^3^, as compared to the value of 860 Å^3^ observed for the parental XynA, demonstrating that the volume of the catalytic site is around 25 % larger in the chimeric xylanase than in the parental enzyme. This calculation suggests that the higher xylanolytic activity observed in the chimera may be the result of increased substrate access to the active site, and is consistent with the reduction in the values of *K*_RBB-Xylan_ observed in the chimeric enzymes (see Table 1).

Overall, the MDS results suggest that the XynA–XBP271 chimera presents a more rigid structure as compared to the parental XynA and, in particular the thumb region demonstrates a significant reduction in flexibility. The binding of xylose to the XBP domain led to an additional reduction in flexibility, not only in the xylanase domain but in the entire chimera structure. Although binding of xylose reduced the movement of the XBP domain, limiting its structure to the closed conformation that is inherently less flexible, correlated movements in the xylanase domain resulted in an increase in the volume of the catalytic cleft, indicating that substrate access to the active site may underly the increase in xylanolytic activity observed in the chimeric enzyme. The allosteric effect in the chimera appears to result from correlated structural changes between the XBP domain and the XynA domain on xylose binding, and the MDS results identify several key residues in the XBP/XynA protein-protein interface that may be involved in the interdomain communication.

## Conclusion

The combination of protein domains is a powerful evolutionary mechanism for the generation of new architectures and new functions in proteins [33]. Here, using a directed evolution approach, we have combined the non-homologous proteins xylanase and XBP and created chimeric protein XynA–XBP271 with increased catalytic efficiency. The heterotropic effector d-xylose was a positive allosteric modulator of the xylanase domain in the chimera. The approach presented here provides an important advance for the engineering enzymes that are stimulated by the final product.

## Methods

### Plasmid construction

The chromosomal region 3168595–3169587 from *E. coli* K12 including the signal sequence and the protein coding sequence of the xylF gene encoding the xylose binding protein (XBP) (Gene ID: 948090, 993 nt) was cloned into the vector pT7T3GFP to construct the plasmid pT7T3GFP_XBP as described previously [17]. The protein coding sequence without the signal sequence from the *xynA* gene from *B. subtilis* (GeneID: 939861, 558 nt) was cloned into the plasmid pT7T3 18U (GeneID: U13869.1; GE Healthcare, Fairfield, Connecticut, USA) using the restriction sites *Hind*III and *Bam*HI, to generate the construct pT7T3/XynA.

### Library creation by random insertion of XynA into XBP

A random insertion library of xylanase in XBP was created using the protocol developed by Guntas and Ostermeier [14, 15] with some modifications. Fifty micrograms of the pT7T3GFP_XBP plasmid was mixed with 50 mM Tris HCl (pH 7.5), 1 mM MnCl_2_ and 50 µg/mL BSA, and the volume was completed to 95 µL with DNase-free water. Five µL of DNase I (Promega) containing 5 mU of the enzyme was then added. The mixture was incubated for 8 minutes at 22 °C, and the reaction was stopped by the addition of 2.4 µL of 0.5 M EDTA and incubation at 75 °C for 10 minutes. The DNA from this reaction was purified and eluted in 200 µL of water, and a sample of 100 ng of DNA was run on an agarose gel to estimate the percentage of linear DNA. The repair step was then performed in which T4 DNA ligase and T4 DNA polymerase (both enzymes from New England Biolabs, Ipswich, MA, USA) were added in a ratio of 160:1 respectively, per 1 µg of linear DNA. The mixture was incubated at 12 °C for 20 minutes with T4 ligase buffer and 200 μM dNTPs. The reaction was stopped by the addition of 10 mM EDTA and heating to 75 °C for 15 minutes. The linear DNA (5041 bp) was then purified using agarose gel. Approximately 1.5 µg of DNA was dephosphorylated using Antarctic phosphatase 12.5 U (New England Biolabs, Ipswich, MA, USA) in Antarctic phosphatase buffer for 30 minutes at 37 °C.

To create the random insertion library, the *xynA* gene without the stop codon was amplified by PCR from the pT7T3/XynA vector using phosphorylated primers and ligated to the gel purified pT7T3GFP_XBP plasmid prepared as described in the previous paragraph. The product of the ligation reaction was purified, concentrated and used to transform electrocompetent kanamycin resistant JW3538-1 *E. coli* cells that lack the XBP gene [the xylF749(del)::kan strain from the Coli Genetic Stock Center, Yale University, USA, hereafter referred to as ΔxylF]. After regeneration, the cells were plated on LB-agar containing 34 µg/mL kanamycin and 100 µg/mL ampicillin, on bioassay plates (245 × 245 cm). After growth, all the bacterial colonies present on the plates were harvested in storage media [LB + 10 % glycerol (v/v)] and stored at −80 °C.

### Screening for the binding activity of XBP

A 500 µL aliquot of cells from the libraries stored at −80 °C was used to inoculate 50 mL of tryptone broth (TB) (10 g tryptone and 5 g NaCl per liter) containing 34 µg/mL kanamycin, 100 µg/mL ampicillin and 10 mM xylose. The culture was grown in an orbital shaker at 250 rpm and 37 °C for 10 h, and the culture was centrifuged. The cell pellet was resuspended in phosphate buffered saline (PBS) to a concentration of ~10^6^ cells mL^−1^, after which the cells were kept on ice. Flow cytometry analysis fluorescent assisted cell sorting (FACS) was performed on a FACSAria cytometer (Becton, Dickinson and Company, East Rutherford, NJ, USA) equipped with a 405 nm excitation laser and a 530/30 nm bandpass emission filter. For each sample, 10^4^ events were collected at a rate of 500–1000 events per second, where data collection and analysis used the FACSDiva software (Version 6.1.1., BD Biosciences, San Jose, CA, USA). Cells transformed with the pT7T3XBP vector were used as the negative control for correction of the auto-fluorescence, and cells transformed with the pT7T3GFP_XBP vector were used as the positive control. Clones that produced higher fluorescence than cells transformed with pT7T3GFP were collected and were denominated as XBP+ clones.

### Screening for xylanolytic activity

The XBP+ clones separated by FACS were plated on selective LB-agar media. After incubation at 37 °C for 12 h, individual colonies were transferred to 384-well microplates containing 60 µL selective TB media using an automated colony picker (model K6, Kbiosystems, Basildon, Essex, UK). The plates were incubated at 37 °C for 24 h, and replicated onto 245 × 245 mm bioassay plates containing TB-agar media, supplemented with 0.6 % (m/v) xylan, 1 % (m/v) xylose; 34 µg/mL kanamycin; 100 µg/mL ampicillin. After incubation of the plates at 37 °C for 24 h, the clones expressing xylanase activity were identified by the formation of halos after staining with Congo red [34], and were denominated as XBP+/XynA+ clones.

### Measurement of xylose stimulated catalytic activity

The XBP+/XynA+ clones were grown in TB supplemented with 34 µg/mL kanamycin, 100 µg/mL ampicillin for 48 h in 96-well plates (deep well). The supernatants were analyzed for hydrolysis of Remazol Brilliant Blue Xylan (RBB-xylan, Sigma-Aldrich, St. Louis, Missouri, USA), using a modification of a previously described protocol [35]. Fifty µL of culture supernatant was mixed with 50 µL of a solution containing RBB-xylan (4 mg/mL) in 100 mM acetate buffer (pH 5.5), in the presence or absence of 1 % (m/v) d-xylose (Sigma-Aldrich, St. Louis, Missouri, USA), and incubated at 37 °C for 12 h. After incubation, the reaction was stopped by the addition of 2 volumes (200 µL) of 96 % (v/v) ethanol. The insoluble material was removed by centrifugation (2000*g*/2 minutes), and the increase in the absorbance of the supernatant was measured at 595 nm. A single clone that showed activity ratio (with xylose)/(without xylose) greater than 1.3 was selected, and nucleotide sequencing indicated that the XynA domain was inserted within the XBP at position 271, and this chimeric enzyme was denominated as XynA–XBP271.

### Expression and purification of the recombinant enzymes

The parental XynA, parental XBP and the chimeric XynA–XBP271 enzyme were expressed in *E. coli* [Rosetta™ (DE3)] transformed with pET28a (+) (Novagen, Billerica, MA, USA) carrying XynA, XBP or XynA–XBP271 with a N-terminal His6-tag and grown in HDM medium containing (per liter) 25 g of yeast extract, 15 g of tryptone, 1.2 g of MgSO_4_, supplemented with 34 µg/mL kanamycin and 40 µg/mL chloramphenicol. The cells were grown at 30 °C/120 rpm to an OD_600_ of 0.6. In all the cases, protein expression was induced with 0.5 mM isopropyl-D-thiogalactopyranoside (IPTG) for 5 h at 20 °C/120 rpm. Cells were harvested by centrifugation (8000*g*, 4 °C, 10 minutes). Whole-cell extracts were prepared from cell pellets by ultrasonication in 4 % (v/v) of the original culture volume of lysis buffer (100 mM HEPES, 300 mM NaCl, 0.5 mM phenylmethylsulfonyl fluoride, 1 % (v/v) Triton X-100, and 20 mM imidazole, pH 7.5). The cell extracts were cooled on ice and cleared of cell debris by centrifugation (10,000*g*, 4 °C, 30 minutes). The supernatants were loaded on an immobilized metal affinity column Ni–NTA (GE Healthcare, Fairfield, Connecticut, USA) pre-equilibrated with a buffer containing 100 mM HEPES, 300 mM NaCl, and 20 mM imidazole (pH 7.5). The column was washed with buffer containing 100 mM HEPES (pH 7.5), 300 mM NaCl, and 40 mM imidazole until no further reduction in the A_280_ was observed. Protein was eluted with 300 mM imidazole, and protein samples were dialyzed against 20 mM Tris–HCl (pH 8.0) and 200 mM NaCl and stored at 4 °C for further use. The protein concentrations were determined by measurement of the A_280_.

### Enzyme activity assays

The effect of pH on xylan hydrolysis by the purified enzymes was determined at 40 °C in 50 mM with 0.2 % (w/v) RBB-xylan substrate (Sigma-Aldrich, St. Louis, Missouri, USA) buffered with one of the following buffer systems: acetic acid/acetate (pH 4.5-5.5), potassium phosphate (pH 5.5–6.5), MOPS-NaOH (pH 6.5–7.5) and Arginine-NaOH (pH 9.0). The effect of temperature on xylanase activity was conducted at temperatures between 30 and 55 °C in 50 mM acetate, pH 5.5. Thermostability was assessed by incubation of the purified enzymes at 55 °C and residual activity was measured in aliquots collected at increasing times. The kinetic parameters for xylanase were determined using the RBB-xylan substrate at concentrations ranging from 0.5 to 10 mg/ml, with and without 1 % (w/v) d-xylose (Sigma-Aldrich, St. Louis, Missouri, USA). The reactions were initiated by the addition of 50 nM of the purified enzyme to MOPS buffer (pH 6.5) at 45 °C. After 15 minutes, the enzyme was inactivated by incubation at 80 °C for 10 minutes, followed by incubation at 4 °C for 5 minutes. One hundred microliters of ethanol were then added and the mixture was incubated at 25 °C for 15 minutes. The samples were centrifuged at 2000*g* for 2 minutes and 90 µL of each sample and transferred to a 96-well plate. The absorbance values were measured at 595 nm and converted to μmols of released dye using a RBB-xylan substrate standard curve generated under the same conditions. All enzymatic activities were determined in triplicate and the maximum velocity (*V*_max_), apparent dissociation constant (*K*_RBB-Xylan_), and catalytic constant (*k*_cat_) were calculated by nonlinear regression fitting of the data to the semi-logarithmic form of the Hill equation using the SigrafW software [36].

### Enzyme assays using sorghum stover

The activity of enzymes on a natural substrate was evaluated using ground sorghum stover, previously washed with 100 mM MOPS buffer (pH 6.5) to remove residual soluble sugars. A 1 % (w/v) suspension of the washed substrate was prepared in the same buffer and mixed with either 30 nmol of the purified chimera, 30 nmol of individual purified xylanase or with an equimolar mixture comprised of 30 nmol of xylanase and 30 nmol of XBP, in a final reaction volume of 5 mL. The reaction was incubated at 40 °C for 15 h in a temperature controlled orbital shaker at 250 rpm to avoid substrate precipitation, and the total reducing sugar release was measured using the 3,5-dinitrosalicylic acid (DNS) assay [37]. All samples were assayed in triplicate and the mean of the three values was used for subsequent comparisons.

### Molecular dynamics simulations and molecular modelling

Molecular dynamics simulations (MDS) and analyses were performed with the XynA–XBP271 chimera, both with and without bound xylose using the GROMACS 5.0.2 software package [38–40] with the GROMOS-96(53A6) force field [41]. The starting atomic coordinates of the chimeras were obtained by comparative protein modeling with program MODELLER 9.13 [42] to merge the XynA (pdb code:1XXN) and the XBP in the open (PDB code: 3M9 W, xylose-free) and closed conformations (PDB code: 3MA0, xylose-bound), respectively. These initial structures were validated using Procheck software [43] with a subsequent energy minimization step using the steepest descent method. The resulting structures were solvated by SPC water molecules at a concentration of approximately 54 mol/L in dodecahedron simulation boxes. Na^+^ ions were added to ensure the electroneutrality of the systems. Position restrained dynamics were performed for 400 ps at a reference temperature of 300 K to improve the equilibration phase. All systems were carried out in the NVT ensemble at neutral pH and 300 K, with a total time simulation of 120 ns. Temperature was controlled by a V-rescale thermostat [44] and covalent bonds involving hydrogen atoms in the protein and water molecules were restrained by LINCS [45] and SETTLE [46] algorithms, respectively. Newton’s equations of motion were solved using the Leap-Frog integration method [47] with dt = 2.0 fs. The Maxwell–Boltzmann distribution at a reference temperature was employed to generate the initial atomic velocities. The particle-mesh Ewald sum (PME) [48] was used to treat the long-range interactions with a 1.2 nm cutoff distance. The interaction potential energy (IPE) can be defined as the total interaction energy between protein A and protein B, and was computed according to the equation:

$$IPE = \sum_{i}^{NA}\sum_{j}^{NB}E_{i,j}$$

where $E_{ij}$ is the interaction energy between an atom $(i)$ from protein A and an atom $(j)$ from protein B, and *NA* and *NB* are the total number of protein A and B atoms, respectively. Computational Alanine Scanning (CAS) was performed using the ROBETTA program [49] to identify “hotspot” residues at the protein–protein interface.

## Additional files

10.1186/s13068-016-0529-7 Screening of the xylose stimulated xylanase/XBP chimera.
10.1186/s13068-016-0529-7 SDS-polyacrylamide gel analysis of the chimeric and parental enzymes purified from recombinant E. coli Rosetta™ (DE3).
10.1186/s13068-016-0529-7 Hot spot residues at the protein–protein interface between the XBP and XynA domains by molecular dynamics simulations.

## Abbreviations

CAZy: carbohydrate-active enzymes database
GH11: glycosyl hydrolase family 11
XynA–XBP271: chimeric enzyme in which the xylanase was inserted after residue 271 of the XBP
pT7T3GFP_XBP: vector used in this work
DNS: 3,5-Dinitrosalicylic acid
PDB: protein data base
IPTG: isopropyl β-D-1-Thiogalactopyranoside
MDS: molecular dynamics simulation
