# Supplementary material for: A xylose-stimulated xylanase–xylose binding protein chimera created by random nonhomologous recombination
Source: Biotechnol Biofuels. 2016 Jun 6;9:119. doi: 10.1186/s13068-016-0529-7 (PMC4896006; doi:10.1186/s13068-016-0529-7)
Supplement: Supplementary file 1 — 10.1186/s13068-016-0529-7 Screening of the xylose stimulated xylanase/XBP chimera. [file 13068_2016_529_MOESM1_ESM.docx]

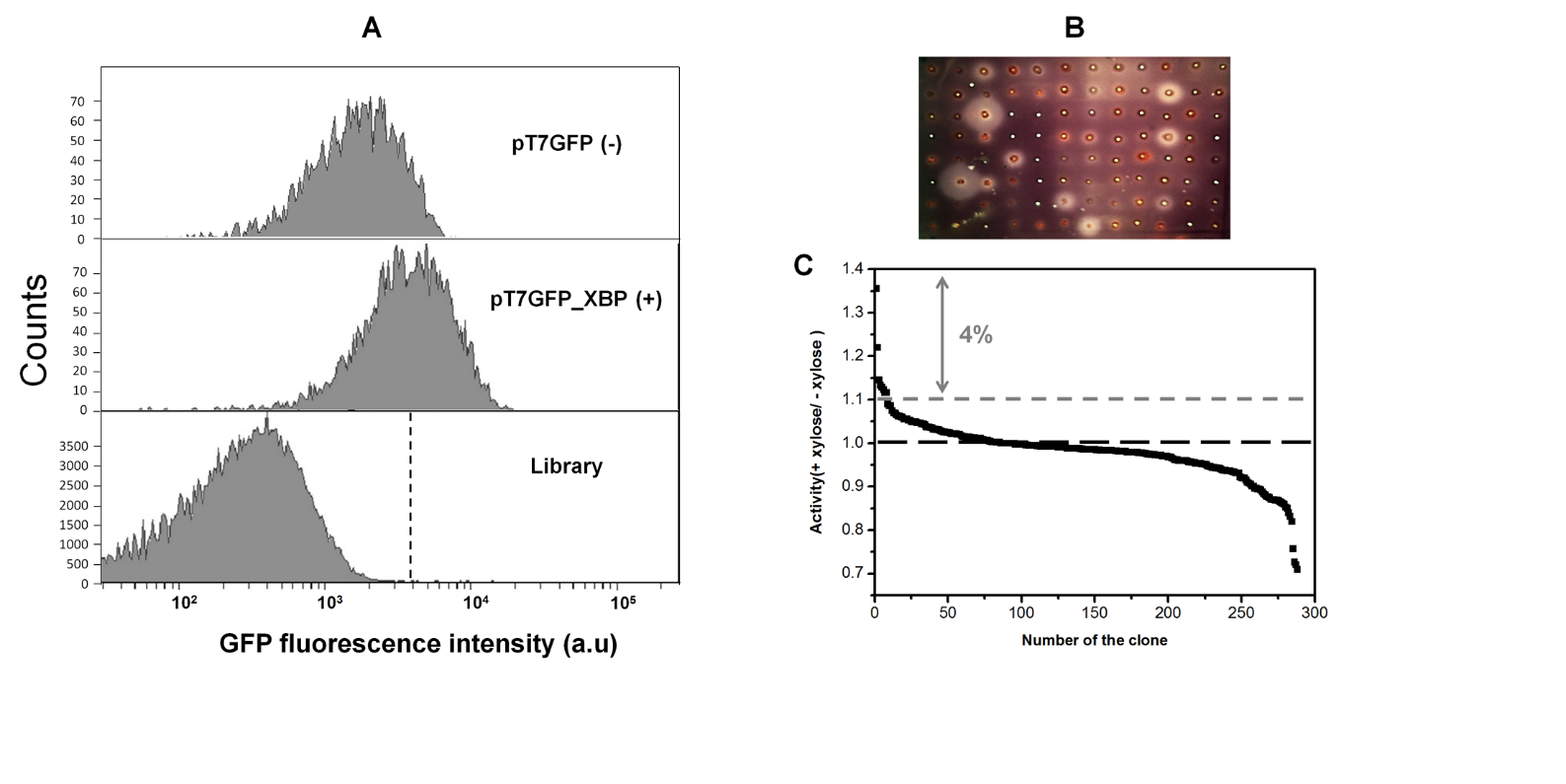


Additional file 1. Screening of the xylose stimulated xylanase/XBP chimera. A) FACS screening in *E. coli* ΔxylF cells of the insertion library created using DNase I in pT7GFP_XBP. Functional XBP permits the cellular capture of extracellular xylose, increasing its intracellular concentration. The expression of GFP is under the control of the xylose inducible promoter P_xylF_. The positive and negative controls (pT7GFP and pT7GFP_XBP) were used to define the limits for the populations XBP+. B) Xylanase activity as detected by halo formation by XBP + colonies on xylan agar plates containing 1% (m / v) D-xylose as detected after staining with a 0.1% (w / v) Congo red solution. C) *In vitro* screening of the XBP+/XynA+ clones in the presence and absence of xylose, plotted in decreasing order. Clones that showed an increase in activity greater that 10% in the presence of xylose (short-dashed line) were selected for further analysis. The long-dashed line shows the limit at which there was no difference in activity in the presence of D-xylose.
