# Supplementary material for: A xylose-stimulated xylanase–xylose binding protein chimera created by random nonhomologous recombination
Source: Biotechnol Biofuels. 2016 Jun 6;9:119. doi: 10.1186/s13068-016-0529-7 (PMC4896006; doi:10.1186/s13068-016-0529-7)
Supplement: Supplementary file 2 — 10.1186/s13068-016-0529-7 SDS-polyacrylamide gel analysis of the chimeric and parental enzymes purified from recombinant E. coli Rosetta™ (DE3). [file 13068_2016_529_MOESM2_ESM.docx]

**
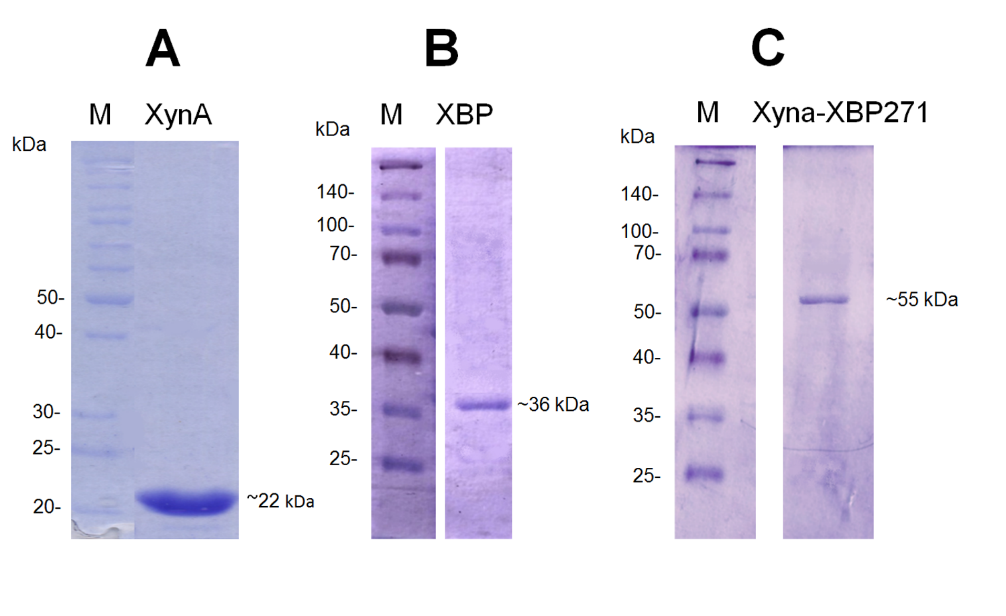
**

**Additional file 2. SDS-polyacrylamide gel analysis of the chimeric and parental enzymes purified from recombinant E. coli Rosetta™ (DE3).** A) parental XynA; B) parental XBP; C) XynA-XBP271 chimera. M: Marker molecular weights are indicated on the left of the gel, and estimated molecular weights of the purified proteins are indicated to the right. The gel was stained using Coomassie Brilliant Blue.
