# Supplementary material for: A xylose-stimulated xylanase–xylose binding protein chimera created by random nonhomologous recombination
Source: Biotechnol Biofuels. 2016 Jun 6;9:119. doi: 10.1186/s13068-016-0529-7 (PMC4896006; doi:10.1186/s13068-016-0529-7)
Supplement: Supplementary file 3 — 10.1186/s13068-016-0529-7 Hot spot residues at the protein–protein interface between the XBP and XynA domains by molecular dynamics simulations. [file 13068_2016_529_MOESM3_ESM.docx]

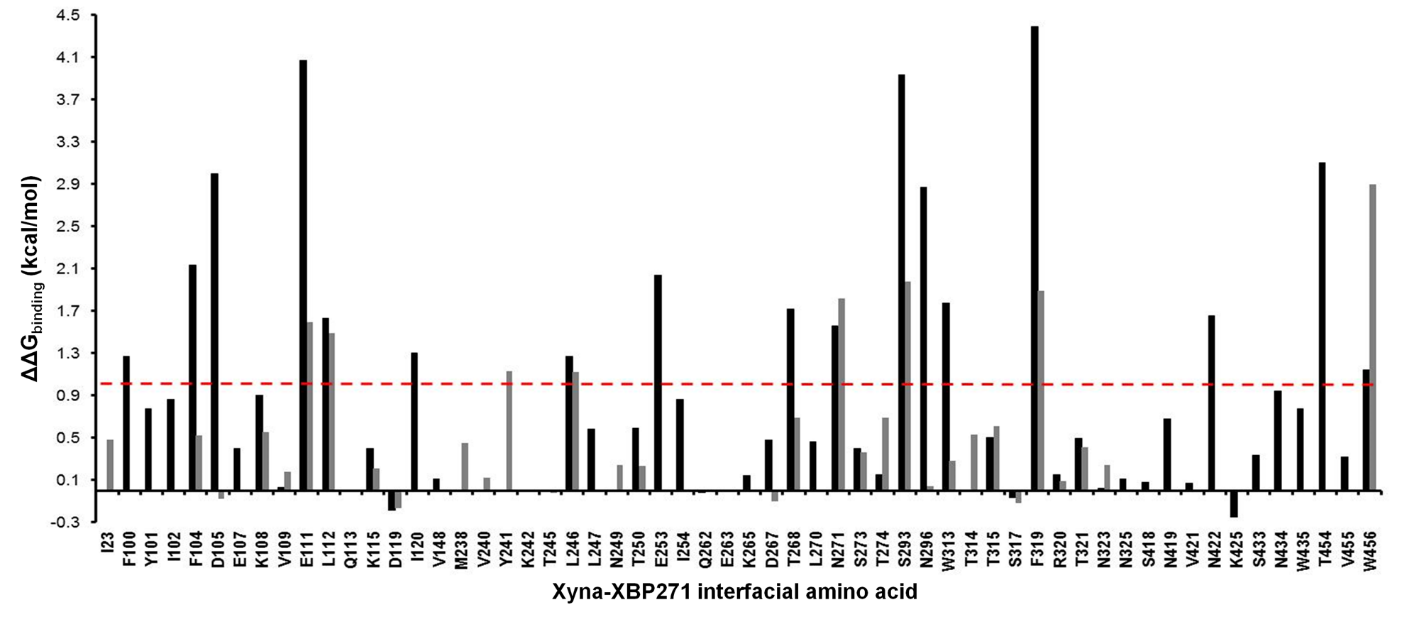


**Additional file 3. Hot spot residues at the protein–protein interface between the XBP and XynA domains by molecular dynamics simulations.** Changes in binding free energy changes (ΔΔG_bind_) after alanine mutation of interface residues in the XynA-XBP271 chimera in the presence (black bars) and absence (gray bars) of xylose. Amino acids with a ΔΔG_bind_> 1.0 kcal/mol (above the threshold shown by the dotted red line) are considered to contribute to hot-spots at the interface.
